# Enhancing antibody folding and secretion by tailoring the *Saccharomyces cerevisiae* endoplasmic reticulum

**DOI:** 10.1186/s12934-016-0488-5

**Published:** 2016-05-23

**Authors:** Jorg C. de Ruijter, Essi V. Koskela, Alexander D. Frey

**Affiliations:** Department of Biotechnology and Chemical Technology, Aalto University, Kemistintie 1, 02150 Espoo, Finland

**Keywords:** Protein folding, Heterologous protein production, Endoplasmic reticulum, Antibody, Chaperones

## Abstract

**Background:**

The yeast *Saccharomyces cerevisiae* provides intriguing possibilities for synthetic biology and bioprocess applications, but its use is still constrained by cellular characteristics that limit the product yields. Considering the production of advanced biopharmaceuticals, a major hindrance lies in the yeast endoplasmic reticulum (ER), as it is not equipped for efficient and large scale folding of complex proteins, such as human antibodies.

**Results:**

Following the example of professional secretory cells, we show that inducing an ER expansion in yeast by deleting the lipid-regulator gene *OPI1* can improve the secretion capacity of full-length antibodies up to fourfold. Based on wild-type and ER-enlarged yeast strains, we conducted a screening of a folding factor overexpression library to identify proteins and their expression levels that enhance the secretion of antibodies. Out of six genes tested, addition of the peptidyl-prolyl isomerase *CPR5* provided the most beneficial effect on specific product yield while *PDI1*, *ERO1*, *KAR2*, *LHS1* and *SIL1* had a mild or even negative effect to antibody secretion efficiency. Combining genes for ER enhancement did not induce any significant additional effect compared to addition of just one element. By combining the Δ*opi1* strain, with the enlarged ER, with *CPR5* overexpression, we were able to boost the specific antibody product yield by a factor of 10 relative to the non-engineered strain.

**Conclusions:**

Engineering protein folding in vivo is a major task for biopharmaceuticals production in yeast and needs to be optimized at several levels. By rational strain design and high-throughput screening applications we were able to increase the specific secreted antibody yields of *S. cerevisiae* up to 10-fold, providing a promising strain for further process optimization and platform development for antibody production.

**Electronic supplementary material:**

The online version of this article (doi:10.1186/s12934-016-0488-5) contains supplementary material, which is available to authorized users.

## Background

The yeast *Saccharomyces cerevisiae* has proved its value in industrial applications, such as small chemicals and biofuel production, and is an excellent choice for new biotechnological processes due to the wide range of techniques to manipulate and cultivate the organism efficiently [1]. *S. cerevisiae* is also a popular choice for production of recombinant proteins and is currently the chosen production host for one-fifth of approved biopharmaceuticals on the market [2]. One of the main limitations of producing recombinant pharmaceuticals in yeast is the different *N*-glycan pattern compared to mammalian expression platforms [2]. However, producing correctly folded and glycosylated proteins for therapeutic applications in *S. cerevisiae* is about to become possible with the advancements of glycoengineered strains [3, 4]. With further optimization of the host and the production process, the yeast has great potential to become a viable platform to produce competitive yields of complex, functional mammalian target proteins, such as antibodies.

The yeast endoplasmic reticulum (ER) is limited in its recombinant protein folding capacity, and is thus an important target for cell engineering to improve product yields [5, 6]. Producing recombinant proteins in yeast often leads to accumulation of folding intermediates in the ER and consequently to induction of the unfolded protein response (UPR) [7]. The UPR is a transcriptional program for concomitant upregulation of over 300 genes, including the upregulation of basal expression levels of constitutively expressed quality control components in the ER, which include folding factors, chaperones and the ER associated degradation (ERAD) machinery [8–10]. Inactivation of the UPR has shown to be detrimental for heterologous protein production [11, 12]. Although the UPR response is shown to be specific to the source of stress [13], heterologous protein yields could benefit most from a protein specific, fine-tuned expression of necessary helper proteins. One of the effects of UPR activation is also the Ino2/4 dependent induction of lipid biosynthesis genes, which play a key role in phospholipid biosynthesis required for membranes [14]. Although UPR leads to ER expansion, in yeast it has been shown that increasing ER size alone is sufficient to alleviate conformational stress independently from chaperone levels and UPR-induction [15]. Importance of ER size in effective protein secretion can also be noted from professional secretory cells, such as plasma cells and thyrocytes, that are characterized by an expanded ER that occupies most of the cytoplasmic space [16–19]. Although the development of secretory cells employs some of the same molecular routes as the UPR and has similar effects on gene expression, the onset of protein production is preceded by ER expansion [20]. Thus increasing the size of ER seems like a sensible strategy to preempt the negative effects of protein overexpression stress and the concomitant induction of UPR.

The limited folding rate in the ER of *S. cerevisiae* becomes a critical factor when producing large and complex proteins, such as human antibodies. In their native production environment, plasma cells, antibodies employ several classes of folding factors in their maturing process [21]. Molecular chaperones, such as BiP and its co-chaperones, and folding enzymes, like protein disulfide isomerases (PDIs) and peptidyl-prolyl-isomerases (PPIase), all interact in a sequential manner with the folding light and heavy chain polypeptides to produce the secreted, mature heterotetrameric antibody [21, 22]. Although the yeast ER contains folding factors from all these classes, it is questionable whether the levels and stoichiometry of the proteins are adequate for efficient IgG-assembly. Different folding assistants co-operate with each other and form an extensive interaction network, possibly even a multiprotein complex [23–25]. Addition of just Pdi1p and/or Kar2p to enhance folding has been a popular approach [6, 26–28], but to obtain predictable effects the stoichiometry between the different folding factors should be considered. Achieving the correct balance of folding factor expression is a laborious task and high-throughput techniques need to be implemented to strain development in order to gain improvements in protein production.

In this study, we demonstrate how yeast protein secretion can be enhanced by increasing the ER space through deletion of the *OPI1*-gene. By using this strain along with the wild-type, we conducted a comprehensive screening of six yeast folding factors under control of up to five different promoters to identify elements and expression levels that were beneficial for IgG secretion. Overexpression was combined with the expanded ER to produce a strain with high secretion levels. Our focus was on modifying the ER environment, and we discuss how the different modifications might affect heterologous protein production in yeast.

## Results and discussion

### Increasing the size of yeast ER increases IgG secretion levels by more than fourfold

The *S. cerevisiae* gene *OPI1* is involved in regulation of lipid-metabolism, and its main target is the Ino2p/Ino4p-complex. Ino2p and Ino4p are transcription factors that work as a heterodimer to induce the transcription of many phospholipid synthesis enzymes [29, 30]. The protein product of *OPI1* functions as a repressor for the Ino2p/Ino4p-complex as Opi1p binds to Ino2p and inhibits the formation of the active heterodimer in lipid abundant conditions [31]. Removal of Opi1p by gene knock-out resulted in a constitutively active Ino2p/Ino4p-complex, which upregulated the *de novo* synthesis of lipids [15, 29, 30]. The Ino2p/Ino4p-complex has a key role in ER size control, and removing its repressor Opi1p causes the yeast ER to expand by approximately 1.5-fold compared to the wild-type yeast ER [15].

Increase in ER size is a characteristic feature of professional secretory cell development [32, 33] and has been stated to reduce ER stress in *S. cerevisiae* [15], thus we decided to study whether increasing ER size before induction of protein production would correlate with an increased IgG yield in our system. We generated a yeast strain that, in addition to having a galactose-inducible cassette for encoding full-length human IgG inserted to its genome, had the *OPI1*-gene deleted. This strain, named here as Δ*opi1*, was used throughout the screening along with the parental W303α-strain that had only the IgG expression cassette insertion, designated here as the wild-type (wt).

**Fig. 1:**
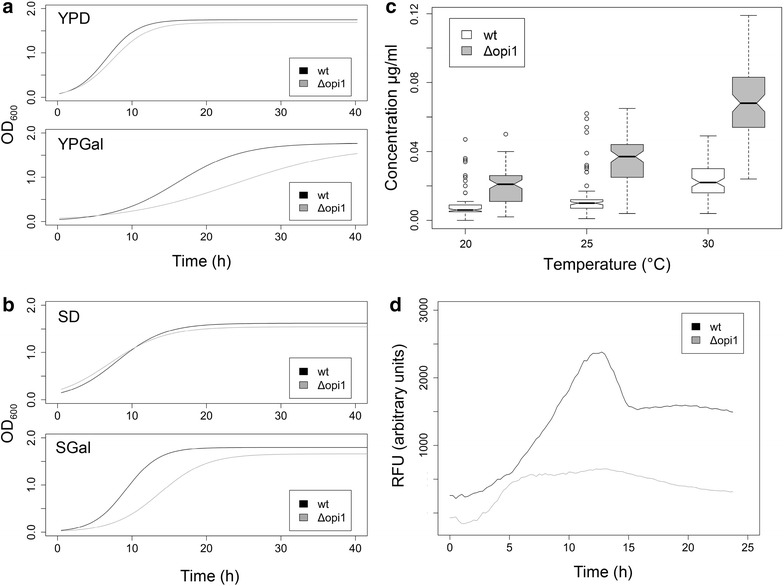
Δ*opi1* deletion increases antibody production through increased ER size. **a** Growth of the two background strains was measured in complex (**a**) and synthetic media (**b**) at 30 °C. Used media are indicated in the *graphs*. *Upper panels* represent growth under non-inducing conditions, *lower panels* growth under inducing conditions. When antibody expression was not induced (**a** and **b**, *upper panels*), growth behavior of wild-type (wt) and Δ*opi1* strains was similar. While antibody expression was induced (**a** and **b**, *lower panels*), growth of Δ*opi1* mutant was reduced. **c** A notched *box-plot* presentation of all the measured antibody concentrations for the background strains shows that antibody secretion was considerably more efficient for Δ*opi1* in all temperatures. The number of measurement points used for each box is 82 or 84. **d** Fluorescence of GFP under an UPR-responsive promoter was recorded in wt and Δ*opi1.* The background corrected RFU-values remain much lower for Δ*opi1*. Highest GFP fluorescence of wt was two times higher per OD compared to Δ*opi1*

Growth of wt and Δ*opi1* strains was compared under non-inducing and inducing conditions for 40 h in complex YPD and synthetic defined media. Under non-inducing conditions, the Δ*opi1*-mutant did not display any defects in growth, but reached a slightly lower optical cell density (OD_600_) in complex media compared to the wild-type, in stationary phase the OD_600_ being 1.762 for wt and 1.698 for Δ*opi1* yeast strain (*P* value< 0.001) (Fig. 1a). The same behavior was recorded in SD media with raffinose as the carbon source (Fig. 1b). However, during IgG expression, the maximal specific growth rate (µ_max_) was diminished and the µ_max_ was 0.110 h^−1^ for wt and 0.065 h^−1^ for Δ*opi1* cells in YPGal (Fig. 1a). The effect on growth rate was not as severe in SGal medium (Fig. 1b). In SGal the maximum growth rates were 0.280 h^−1^ for wt and 0.266 h^−1^ for Δ*opi1.* However, in the production conditions represented in Fig. 1b, the lag-time of Δ*opi1* was prolonged. Despite generally lower OD_600_ values in the cultures, the mean antibody titer of Δ*opi1* strain was up to three fold higher than that of the wild-type strain in the same conditions (Fig. 1c, Additional file 1). When the antibody titer was normalized to cell density, Δ*opi1* performed up to 4.8-fold better than the wild-type in IgG-secretion when grown at 30 °C (Table 1, Additional file 1), clearly indicating that the characteristic increase in ER size by *OPI1* deletion is beneficial for IgG production. A similar positive effect of ER size manipulation has been reported for membrane protein production where the rerouting of metabolic fluxes from storage lipids to phospholipid biosynthesis led to an increase in ER membrane and was accompanied by an improvement of membrane protein accumulation due to the increased folding space [34]. In addition to increasing the ER functional space and affecting UPR-response, deletion of the *OPI1*-gene might also be beneficial for protein production because of the possible roles of its downstream effectors Ino4p and Ino2p in protein biosynthesis and transport [29], implying that Opi1p could also influence the protein secretion pathway through other, more complex effects.

**Table 1:** List of strains with the highest specific antibody product yields

Strain number	Added elements	Strain	Specific product yield (µg ml^−1^OD600^−1^)	Relative frequency^a^	P value^b^	Fold-change relative to wild-type^c^
154	*CPR5*-*P*_*GPD*_	*Δopi1*	0.2027	0.2	0.000002	10.22
157	*CPR5*-*P*_*KAR2*_	*Δopi1*	0.1992	0.15	0.000159	10.15
155	*CPR5*-*P*_*GAL1*_	*Δopi1*	0.1579	0.1	0.000560	7.99
153	*CPR5*-*P*_*TEF*_	*Δopi1*	0.1525	0.075	0.000036	7.66
156	*CPR5*-*P*_*PDI1*_	*Δopi1*	0.1424	0.05	0.007624	7.15
170	*ERO1*-*P*_*GAL1*_	*Δopi1*	0.1226	0.05	0.020502	6.13
212	*SIL1*-*P*_*GAL1*_	*Δopi1*	0.1223	0.05	0.021128	5.53
162	*PDI1*-*P*_*TEF*_	*Δopi1*	0.1208	0.075	0.279237	6.09
206	*LHS1*-*P*_*GPD*_	*Δopi1*	0.1163	0.05	0.299870	5.88
402	*LHS1*-*P*_*KAR2*_ + *SIL1*-*P*_*KAR2*_	*Δopi1*	0.1139	0	0.011316	5.73
83	None	*Δopi1*	0.0886	0.075		4.47
73	None	wt	0.0199	0		1

The data represent the mean value of the experiments conducted with 0.5 and 2 % galactose induction at 30 °C

^a^Relative frequency in the 95th percentile of *Δopi1*-strains

^b^Calculated with the nonparametric equivalent of t test, Wilcoxon signed rank test using the *Δopi1*-strain without added elements as the reference

^c^Averages of the fold-changes relative to wild-type

Opi1p inactivation was shown to have a direct effect on alleviating conformational stress [15], so we investigated the magnitude of the unfolded protein response under antibody expressing conditions in Δ*opi1* and wt (Fig. 1d). We integrated an UPR-responsive cassette to both strains, which expresses an intracellular GFP-signal upon induction of UPR [35]. Interestingly, in the beginning the increase of signal from the UPR-reporter occurred at the same rate in wt and Δ*opi1*, but in the latter, signal increase persisted only during short time frame (Fig. 1d). As we used an intracellular reporter, growth might have an effect to the results. Nevertheless, if OD_600_ values are taken into account, during the height of UPR-induction levels in wt, the strain showed a much higher UPR-GFP signal than in Δ*opi1* (Fig. 1d, around 13 h). It is possible that the initial UPR-induction occurs at the same strength in wt and Δ*opi1*, but the stress caused by the unfolded proteins is resolved faster in cells with a pre-enlarged ER. The UPR-signal persisted in wt until stationary phase of growth was reached, after which the reporter signal faded to a stationary level (Fig. 1d).

### High-throughput screening set-up for optimizing IgG production in yeast

**Fig. 2:**
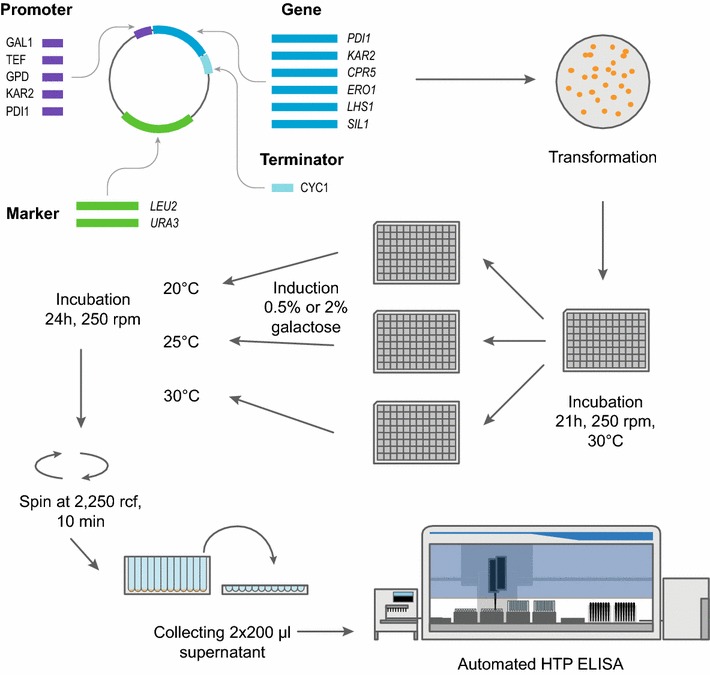
Schematic representation of the screening set-up. We created a plasmid library by making different combinations of three DNA fragments: five different promoters, six coding regions for our selected proteins and two types of vector backbones, all containing the cyc1-terminator and either *LEU2* or *URA3* marker for recombinant selection. Plasmids from the library were transformed into the two background strains, wild-type and *Δopi1*, both having a single-copy of light and heavy chain genes under *GAL1*-promoter integrated into the *HIS3* locus of their genome. Strains were inoculated from solid media to 1 ml of liquid media in 96-deep well plates, in which the strains were precultured for 21 h at 30 °C. The preculture was divided into three plates with fresh media and grown for 5.5 h at 30 °C before antibody expression was induced with 0.5 or 2 % galactose. Antibody was expressed for 24 h at the specified temperatures (20, 25 and 30 °C). Final OD_600_ of cultures was measured. Antibody titers were determined from cleared culture supernatants using two technical replicates with a high-throughput (HTP) ELISA on an automated liquid handling station. Each strain was analyzed in three independent biological replicates

Adjusting the host organism for protein production is a complex process that requires optimization at several levels. The effects of single gene additions are hard to predict and are highly dependent on the client protein [5, 6]. To improve *S. cerevisiae* as an IgG factory, we set up a screening scheme summarized in Fig. 2. We created a plasmid library of six factors that participate in protein folding, and to fine-tune their expression levels, we selected five promoters of different strength to diversify our expression library. The promoters included the constitutive promoters P_GPD_ and P_TEF_, the galactose-inducible GAL1-promoter P_GAL1_, which activates the expression of the folding factor along with the induction of client protein expression, and also two UPR-inducible promoters from yeast genes *KAR2* and *PDI1,* P_KAR2_ and P_PDI1_ respectively, which differ in their basal and inducible expression levels [36]. Each promoter-gene combination was tested both in wild-type and Δ*opi1* strains expressing human IgG. Based on the performances of the strains transformed with a single plasmid of the expression library, plasmids encoding proteins of different functions were combined for another round of testing. In total, we tested 52 individual gene-promoter-strain combinations, and 67 strains with two different gene-containing plasmids.

For initial testing of single elements, plasmids encoding the target genes were cotransformed with an empty plasmid with the complementary marker. This allowed us to use exactly the same media for initial testing and testing of combinations, as every strain contained both *URA3* and *LEU2* auxotrophic markers. The two background strain were transformed with two empty plasmids. In addition, this approach avoided bias in growth and metabolism, as different plasmid complementation has been shown to have an effect on various cellular processes [37]. All screening experiments were conducted entirely in 96-deep well format, inoculating a colony from solid media to one ml of liquid media in each well. As shown in Fig. 2, after the preculture the cells were divided into three replicate plates. Replica plates were grown at 20, 25 and 30 °C. For induction of IgG expression, galactose was added to final concentrations of 0.5 and 2 %. Each strain was cultivated three times, with inoculation of a different transformant and variation of the position in the culture plate to minimize plate position bias. The multiplexed conditions allowed us on one hand to gather better understanding about the link between a particular strain and antibody production and on the other hand on the effect of culture condition on IgG production. For analysis, OD_600_ was measured, and clarified supernatants were collected in duplicates from deep-well plates to determine the antibody titer with automated ELISA.

### Antibody secretion increased with temperature and the effect of *OPI1*-gene deletion was preserved in all transformant strains

**Fig. 3:**
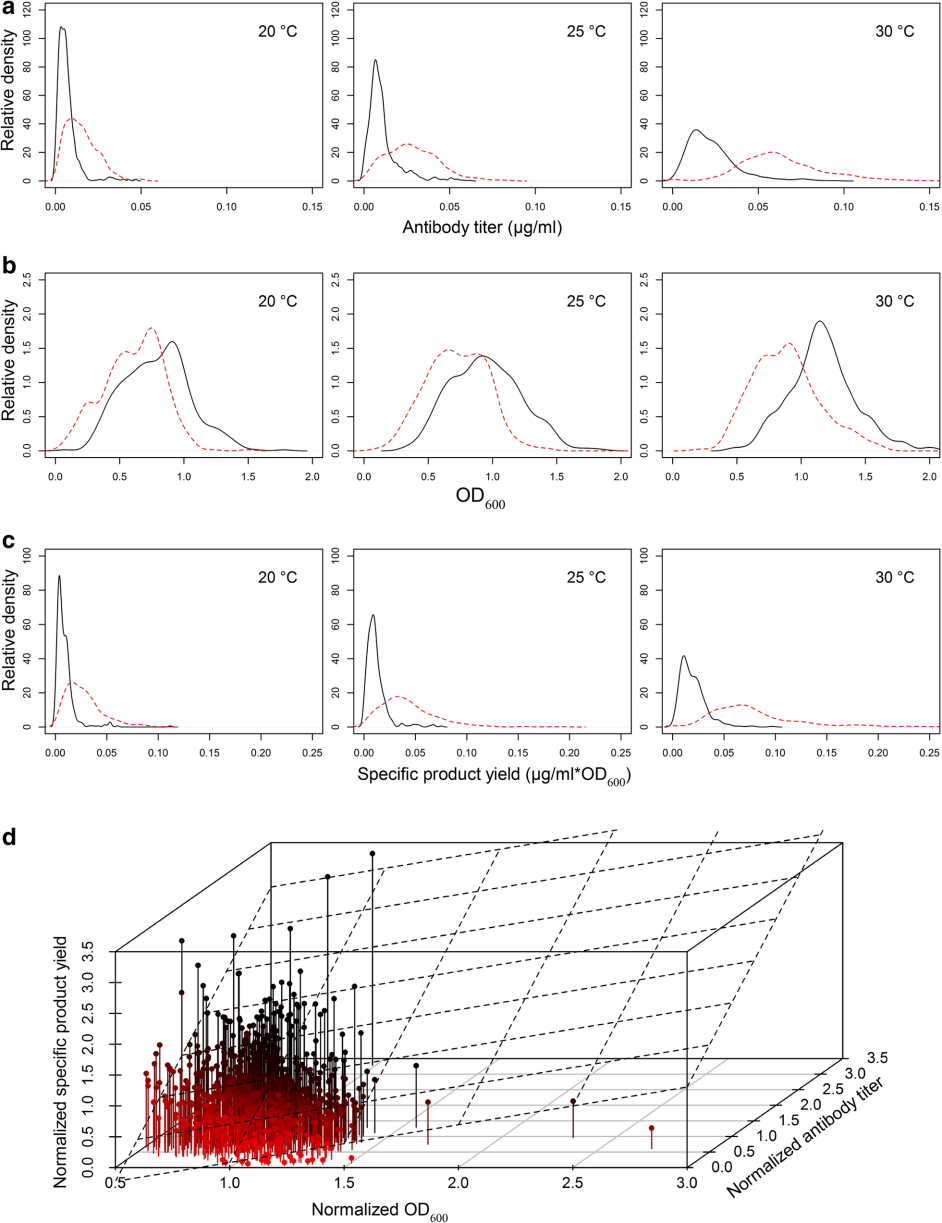
Population analysis of total screening data. Even with added genes, the two background strains perform differently as determined with antibody titer (**a**) and optical density (**b**), forming two populations of measurement values. Δ*opi1* (*dashed red line*) had generally higher antibody concentrations (**a**) and lower OD_600_-values (**b**) than the wild-type (*black solid line*) at all temperatures tested. Distribution of the specific product yields calculated separately for each sample and replicate are shown in (**c**). The distribution of values is shown as density plots, where the area under the line is equal to 1 (total probability of a measurement value being under the line) and the y-axis is proportional to relative frequency. The range of the measured values is given in the x-axis. Each condition included the analysis of 113 different strains and the number of measurement points in each curve is between 798 and 824 in (**a**) and 399–412 in (**b**) and (**c**). The mean values for each strain were normalized to the respective background strain and plotted together to represent all the measured values (**d**). A linear regression plane (*dashed lines*) was fitted to the data points to model the dependency of antibody titer on combined effects of OD_600_ and normalized specific product yield. Multiple R-squared was equal to 0.8896 indicating that a linear dependency was found. The estimated coefficient of fold-change was higher than that for the normalized OD_600_, meaning that a change in specific product yield had a higher impact on the antibody titer values. *Colors* are added only to aid the visual clarity in (**d**)

Throughout all conditions screened, the two genetic backgrounds manifested as two distinguishable populations, as evidenced by differences in antibody production (Fig. 3a), and growth (Fig. 3b). The corresponding distributions of specific product yields of the biological replicates are represented in Fig. 3c and individual replicates expand up to 0.3 µg ml^−1^ OD_600_^−1^. The antibody production increased with temperature (Fig. 3a) along with the OD-values (Fig. 3b), as specific product yield increased to a lesser extent (Fig. 3c). The Δ*opi1* strains reached generally higher antibody titers and lower OD_600_ values manifesting in higher specific product yields, as also was shown for the background strains in Fig. 1. In the Δ*opi1* strains the effects of the added elements were mainly similar as in wild-type, but the relative changes in specific product yield were generally smaller when compared to the wt strain background (Additional files 1, 2, 3). It has been reported that ER expansion in Δ*opi1* strain does not affect the intrinsic cellular levels of ER chaperones, so the functional capacity of ER, defined as the amounts of chaperones and other folding assistants, would not increase according to the size [15]. This might indicate that a diluted ER environment is less prone for IgG aggregation and thus less sensitive to the effects of chaperone overexpression. On the other hand, the clear improvement in antibody secretion achieved with *OPI1*-gene deletion was preserved also during chaperone overexpression (Fig. 3c; Additional files 1, 3).

In total, we identified 49 cases where a more than 50 % increase in antibody titers was measured and 71 cases where a more than 1.5-fold increase of the specific product yield was recorded when compared to the respective strain background. One question that arose from these experiments was whether the higher antibody titers were due to the increased growth or to increased specific product yield. To estimate this, we fitted a linear model to all data points (Fig. 3d). The mean values of OD_600_ and antibody titer from each strain were normalized to the respective background strain values to fit all the conditions to the same value space. A linear regression plane was fitted to the data points to model the dependency of the normalized antibody titer on the normalized specific product yield and normalized OD_600_. The multiple R-squared value for the model was 0.8896, indicating that there was a significant linear dependency. The model estimated the coefficients to be 0.6114 for OD_600_ and 0.8573 for the specific product yield, indicating that improved specific product yield has a bigger positive effect on antibody titer than an increase in OD_600_. The linear model implies that the effects of the elements on secreted antibody amounts are more dependent on changes in cellular productivity than on effects in growth. The calculated P values for the model fit measured by *F* test and also for the coefficients are ≪ 0.001 representing a reliable statistical significance for the linear model.

Both OD_600_ and antibody titer values increased steadily with the temperature (Fig. 3a, b), and the absolute differences between different strains were most prominent at 30 °C. The differences manifested themselves by a wider range of antibody titers at 30 °C (Fig. 3a) and the increasing variance in the strain antibody titer indicates (Additional files 1, 3), that the strain phenotypes diverge more with increasing temperature and thus are more clearly visible at 30 °C. The induction strength also had effect on the IgG secretion, mainly secreting higher amounts at 0.5 % galactose (at 30 °C, mean antibody concentration was 0.0254 µg ml^−1^ for 0.5 % galactose and 0.0207 µg ml^−1^ for 2 % galactose for all strains with wild-type background). When antibody concentration was normalized to OD_600_, the specific product yield did not differ significantly for the two induction conditions (respective values are 0.0210 and 0.0201 µg ml^−1^OD_600_^−1^ for 0.5 and 2.0 % galactose, respectively). Since the two induction strengths resulted in similar outcomes, we focus on 0.5 % galactose in our analysis as it yielded higher concentrations of secreted IgG. The results from all strains tested along with the two background strains are reported in Additional files 1, 3.

### The peptidyl-prolyl isomerase *CPR5*, a novel player to increase antibody production

**Fig. 4:**
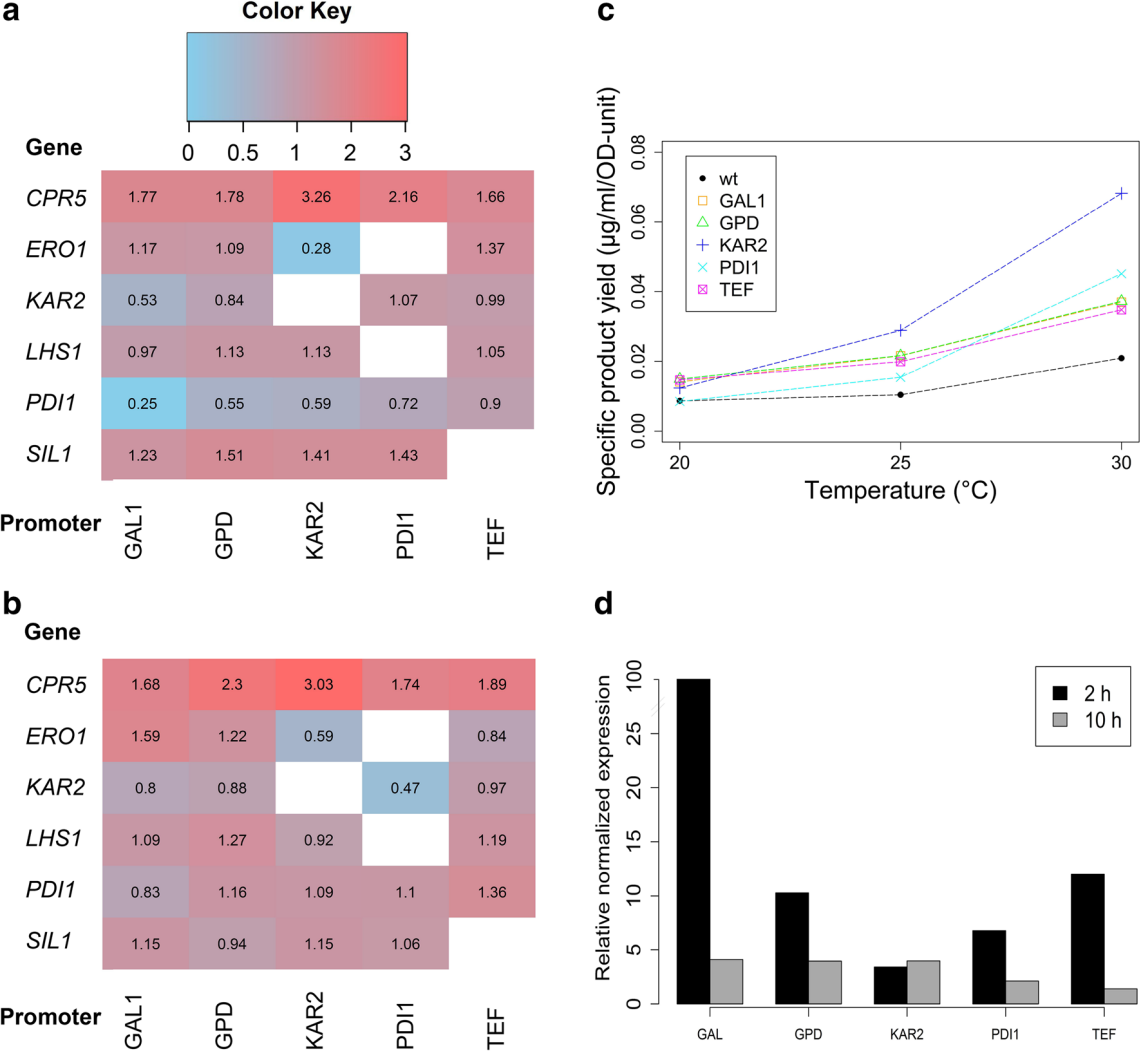
Effects of single folding factor overexpression on antibody secretion. A heatmap representing the change in specific product yield of each strain relative to the strain background. The *colors* and *numbers* in each cells represent the fold-change that the specific promoter gene combination had on the specific product yield. *CPR5* overexpression yielded the most beneficial impact. The values are for wild-type yeast strains grown at 30 °C and for 0.5 % galactose induction (**a**) and Δ*opi1* strains in the same conditions (**b**). **c** Temperature and type of promoter affect the effect of *CPR5* on the specific product yield in wild-type. UPR-controlled promoters, P_KAR2_ and P_PDI1_, show an increase in the positive impact on antibody secretion with increasing temperature, as displayed by the trend line. The specific product yield of wild-type is included as comparison. The values are from measurements with 0.5 % galactose induction. **d** Promoter strengths measured by qPCR of *CPR5* mRNA levels at 2 and 10 h after induction with 2 % galactose at 30 °C. Relative normalized expression is calculated as setting the *CPR5* mRNA levels from wt as control. The promoters show different characteristics, as the UPR-inducible promoters follow the wt levels and P_GAL1_, P_GPD_ and P_TEF_ have higher initial mRNA amount

Antibody titers/specific product yields varied according to chaperone or helper protein, but also according to the promoter used. Figure 4a presents a heatmap that displays a summary of the fold changes in specific product yield that the added elements induced in the wild-type strain and Fig. 4b is a similar heatmap for the Δ*opi1* strain, both at 30 °C with 0.5 % galactose induction. For comparison, heatmaps from 2 % galactose induction are included in Additional file 2, displaying that in general, the effects of the elements are similar in different strain backgrounds and conditions.

Standing out as the most beneficial gene for increasing antibody titers, *CPR5* expression improved the specific product yield 1.65–3.25-fold depending on the promoter and condition used. Cpr5p is an ER localized PPIase involved in the rearrangement of peptidyl-prolyl bonds in unfolded proteins [38]. PPIases have often been neglected in studies of recombinant protein folding and production, although specifically with full-length antibodies, the isomerization of certain proline residues has been determined to be the rate-limiting step in antibody folding in some cases [21, 39–41]. Feige et al. reported also that immunoglobulin domain folding in vitro could be enhanced by addition of the cytoplasmic yeast PPIase Cpr6p [41]. In our study, the best results of *CPR5* expression were achieved with the UPR-induced promoter P_KAR2_, with which the strains showed to be secreting the most IgG at 30 °C (Fig. 4b). As seen in Fig. 4c, the effects of the individual elements were not equal at the different temperatures, as also can be stated for other elements, but generally the induced changes in specific product yield were to the same direction (Additional files 1, 3).

The nucleotide exchange factors Sil1p and Lhs1p are regulating Kar2p activity by maintaining its ATPase cycle through ATP hydrolysis and subsequent ADP release [42, 43]. In Fig. 4a, b we show that using the Kar2p co-chaperones was successful in improving antibody titers, Sil1p showed a clear positive effect (up to 1.5-fold higher secretion), especially in wt background (Fig. 4a), and Lhs1p induced a modest increase in titers. These co-chaperones have been used before to improve the overexpression of human transferrin and recombinant human albumin, although with slightly lower fold-changes [44].

Although overexpression of Pdi1p and Kar2p have been shown to improve the secretion of single-chain antibodies in *S. cerevisiae* [26–28], in our analysis both of these proteins displayed mostly negative effects on antibody secretion at all induction levels and expression conditions (Fig. 4, Additional files 1, 2). The considerable reduction in IgG secretion when overexpressing *PDI1* under strong promoters (P_GAL1_, P_GPD_) was rather surprising, as its overexpression has shown to be increasing titers for various heterologous proteins [45–47]. Besides a possible disruption of the cellular redox balance by a too high concentration of Pdi1p [48], there has also been evidence that the protein facilitates the ERAD of some substrates [49]. The importance of oxidative balance can be also seen from the effects of Ero1p, another redox-active protein, as the secretion of IgG shows to vary greatly depending on the expression level of *ERO1* (Fig. 4a, b; Additional file 2).

Under normal conditions, Kar2p associates with Ire1p in the ER and so represses UPR-induction. When unfolded proteins are present, Kar2p releases from Ire1p and binds them, after that Ire1p can dimerize and induce the UPR [8, 50]. It has been shown that overexpression of Kar2p attenuates Ire1p activation [51] and this makes it possible that the overexpression leads to decreased IgG secretion through an inhibition of the UPR and thus accumulation of the unfolded IgG in the ER. Our data above showed that it is more reasonable to concentrate on regulating the activity of Kar2p by the expression of its co-chaperones Sil1p and Lhs1p, as improved Kar2p cycling might benefit the folding process. It has been estimated that Kar2p is by far the most abundant protein of this trio, with around 300,000 molecules per cell, whereas of Sil1p and Lhs1p around 2400 and 140 molecules are present in the ER [52]. This indicates that increasing the abundance of the regulators can have great effects on Kar2p function. For the production of IgG, this is further supported by data that the Kar2p mammalian homolog BiP does not cycle away efficiently from both unassembled heavy and light chain [53, 54], which might stall protein folding at high Kar2p levels in the ER.

To estimate the extent of chaperone overexpression, we measured the mRNA levels of *CPR5* with qPCR from wt strains overexpressing *CPR5* under control of different promoters 2 and 10 h after induction (Fig. 4d). Compared to the wt strain harboring only the native gene copy under control of the endogenous promoter, *CPR5* expression under control of *GAL1*, *GPD* and *TEF* promoters resulted in a very high initial induction of *CPR5* expression, especially under *GAL1*-promoter (Fig. 4d). Ten hours after induction, *CPR5* mRNA abundance was comparable in all strains harboring an overexpression plasmid. Interestingly, expression of *CPR5* under control of P_*PDI1*_ or P_*KAR2*_ was only slightly enhanced at both time points compared to the control (Fig. 4d). The data indicates that using UPR-responsive promoters enables a moderate overexpression that stays more consistent throughout the production process. The UPR-induced promoters proved to be promising and should be considered when chaperones are overexpressed to enhance heterologous protein production.

### Combining different genes provided little additional benefit to IgG production

After evaluating all the individual elements and determining the best promoter for each gene, we started screening for gene combinations that would further improve IgG secretion. For selecting the combinations, a rational approach was applied based on the known functionalities and interactions of the genes aiming to achieve complementary actions. Optimal expression levels of the gene pairs are crucial but most likely hard to predict, so for the selected pairs we tested a few promoter options. All the selected pairs along with all the measurement results from the screening process are listed in detail in Additional file 3, while Additional file 4 provides a summarized overview of the mean fold-changes. The measurements from the second round are included in Fig. 3, thus the general trends discussed above apply to double gene combinations as well. Again we focus mainly on the results of the experiments performed at 30 °C, as in these conditions the highest antibody titers were obtained and thus the conditions are most interesting for further applications.

**Fig. 5:**
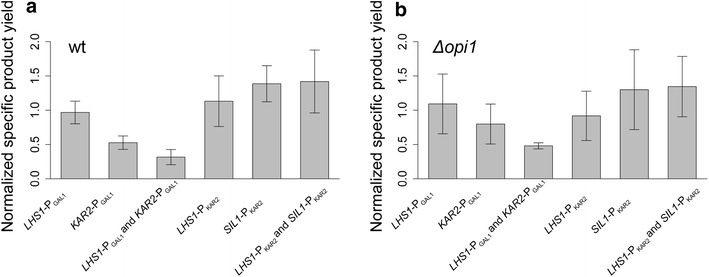
Performance of two-gene combinations compared to the overexpression of only one of the genes. *Bar plots* showing the relative specific product yield for selected gene combinations and the respective single genes in wild-type (**a**) and Δ*opi1* yeast strains (**b**). *LHS1*- P_GAL1_ and *KAR2*–P_GAL1_ seem to have synergistic negative effect since their combination decreases the specific product yield below the level of the combined or individual effects. *LHS1*–P_KAR2_ with *SIL1*–P_KAR2_ had the most positive effect on specific product yield out of all the double gene combinations, but the increase was at the same level as achieved by insertion of *SIL1*- P_KAR2_ alone. Although the effects of the added genes are similar for Δ*opi1* (**b**), the effects are higher in magnitude in wild-type strain background (**a**). *Error bars* represent the standard deviation and the selected values were obtained with 0.5 % galactose induction at 30 °C. Specific product yield is normalized to the mean specific product yield of the respective background strain grown in the same conditions

We did not discover any gene pairs with beneficial synergistic or combinatorial effects. When improving, the fold-changes in overexpression of two elements were less than achieved with just one element. For example, *CPR5* expressed alone induced a significant increase in IgG secretion, but combining the overexpression with other elements, for example *LHS1* or *SIL1*, resulted in no more than 2.3-fold increase at 30 °C (biggest fold-change with *CPR5*-*P*_*KAR2*_/*LHS1*-*P*_*KAR2*_ combination, see Additional file 3), lower than the observed three-fold for *CPR5* alone. As shown in Fig. 5, negative synergistic effects did also occur, often with Kar2p overexpression. The poor performance of two element combinations is reflected by their high occurrence in the lowest, 5th percentile of measurement points (Additional file 5). Targeting of multiple proteins to the ER might be too demanding for the cell, resulting in protein production stress and high occupancy in the translation/translocation machinery thus leading to inefficient secretion. Also, with the overexpression of more folding factors, there is a higher chance to disturb other cellular processes, which might have an indirect combinatorial negative effect on antibody titers. However, overall we did not observe any clear changes in growth when compared to the strains that express only a single element, indicating again that the secretion levels are more dependent on the ability of cells to process IgG and not on changes in OD-values as described in Fig. 3d.

Co-overexpressing Lhs1p and Sil1p, both under P_*KAR2*_, increased the specific product yield by up to 1.5-fold (Fig. 5, Additional files 3, 4). As Lhs1p and Sil1p control Kar2p activity by determining the rate of the ATP-phosphorylation cycle [42], overexpressing both of them might be important to adjust the speed of the Kar2p binding cycle to the increased demand for substrate folding. Although *LHS1*-P_*KAR2*_*/SIL1*-P_*KAR2*_ was one of the best combinations in enhancing the specific product yield, an effect of the same magnitude was achieved by overexpressing Sil1p alone. This indicates that Sil1p is the main protein providing the ADP release step in Kar2p cycling. Interestingly, as with *CPR5* overexpression, the higher antibody titers of the *LHS1*-P_*KAR2*_*/SIL1*-P_*KAR2*_ were accompanied by a slightly lower OD-values (Additional files 3, 4). There might be significant competition for cellular resources between high level protein production and growth [55–57], which might also explain why addition of multiple folding factors did not produce higher antibody titers.

### The strain with highest specific product yield, *Δopi1* + *CPR5*-P_GPD_, has 10-fold higher specific IgG yields than the wild-type

Overexpression of Cpr5p under any promoter proved to yield the highest amounts of secreted IgG per cell. When combined with the *Δopi1*-mutation, the specific product yield could be increased up to 10.2-fold compared to the wild-type in the same conditions. The ten strains with highest mean specific product yields are listed in Table 1. Based solely on 0.5 % induction, as shown in Fig. 4b, *CPR5*-P_KAR2_ provided most enhancement with the *Δopi1*-strain. However, when evaluating a combination of the 0.5 and 2 % conditions, overexpression of *CPR5* under the GPD-promoter provided the highest mean improvement in specific product yield. The *Δopi1* + *CPR5*-P_GPD_ strain yielded specific antibody titers of around 200 ng ml^−^1 OD_600_^−1^, corresponding to a final antibody concentration of 126 µg l^−1^ with 0.5 % galactose induction which is five times more than with the wild-type strain (Table 1; Additional file 1). Although our yeast system was significantly improved, the final antibody concentrations we achieved remained very modest. Many mammalian systems and cell lines, such as Chinese hamster ovary (CHO), HEK293, and a new cell line called F2N78 [58–60], achieve titers in the scale of hundreds of milligrams per liter after unprecedented cell line selection and engineering efforts. Our titers are several magnitudes lower, but comparing different systems is not straightforward in terms of parameters such as final concentration. Performance of different platforms in recombinant protein production was studied by Maccani et al. [61], where the authors show that based on the space–time yield of a single chain antibody, CHO cells perform only 10 times better than the yeast *Pichia pastoris.* Our approach of expressing full-length antibodies differs from the common use of single chain antibodies as the client protein, so it is expected for our variant to display lower yields due to increased complexity. A fivefold increase in space–time yield was obtained here by optimizing just one aspect (folding and ER environment). There are still many approaches left for improving strain performance and yields including many genetic targets and selection of a strain background more suitable for protein production. Together with process optimization, final antibody concentration could reach economically feasible levels in the future.

In Table 1, we display the mean values for the specific product yield at 30 °C for the two induction strengths, as combining these two conditions gives a more reliable estimation for the quantitative change for each element. Within some strains, we observed a high variance between measurements, especially at high antibody titers, probably resulting from a combined effect of differences in cellular state and unavoidable technical inaccuracy (high SD values in Additional files 1 and 4, also Fig. 5). To validate the results of the most different phenotypes, we conducted frequency analysis to determine which strains were enriched among the lowest (5th percentile) and highest (95th percentile) five percent of all measured specific product yields, divided by strain background and temperature (Additional file 5). Representing some outlier measurements, a few instances from the wild-type background strain occurred in both percentiles, since at least one replicate was grown on each deep-well plate to assess the overall success of the plate culture. Many of the same elements occur in the same percentiles in both background strains and different temperatures, indicating that the changes from the elements are generally consistent throughout the assay. Another measure of the reliability of the fold-change of the added elements is the low P-value (as determined by Wilcoxon signed rank test) calculated for many of the strains when compared to the respective strain background. Combination of low P value and high frequency displays a statistically significant fold change and very reliable results for the five best strains with overexpressed Cpr5p, and to most other distinctive phenotypes that we encountered (Table 1, Additional file 5).

### Analysis of cellular IgG clearance from selected strains

**Fig. 6:**
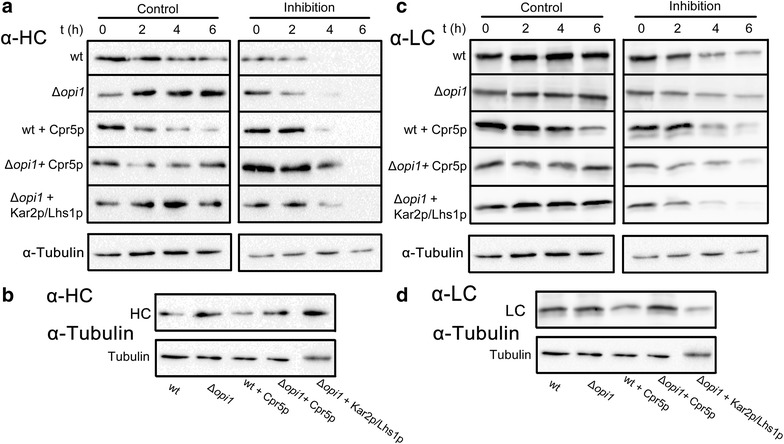
Analysis of cellular IgG clearance using Western blot. **a** Antibody expression in exponentially growing cultures of different strains (wt, *Δopi1, Δopi1* + *CPR5*-P_KAR2_, wt + *CPR5*-P_GPD_ and *Δopi1* + *KAR2*-P_GAL1_ + *LHS1*-P_GAL1_) was induced with galactose for 4 h, after which IgG expression was repressed by addition of glucose, in control experiments, the induction was continued. Heavy chain (HC) clearance was followed for 0, 2, 4 and 6 h. After incubations, cell extracts were prepared from the cultures and analyzed using SDS-PAGE and Western blot. A representative blot for tubulin, used as loading control is shown. **b** Samples from time point zero from all strains were loaded on one gel, and the measured HC signals were visualized using Western blot, their respective tubulin signals are shown as loading controls. **c** Same as in (**a**), but samples were reprobed to visualize the light chain (LC) signals. **d** Same as in (**b**), but samples were reprobed to visualize the LC signals

When using cellular engineering to improve heterologous protein production, it is also useful to gain understanding of the underlying changes in strains with interesting secretion phenotypes. To gain more insight in how some of the overexpressed folding factors influence the progression of the antibody through the cell, we selected five strains for use in a cellular clearance assay as described in De Ruijter and Frey [12]. The strains were as follows: the two genetic strain backgrounds; wt and *Δopi1,* the highest producers from both backgrounds; wt with the *CPR5*-P_KAR2_ plasmid, and *Δopi1* with the *CPR5*-P_GPD_ plasmid, and finally one of the strains with a negatively affecting gene combination: *Δopi1* with the *KAR2*-P_GAL1_ and the *LHS1*-P_GAL1_ plasmids. In short, cultures in the log-phase were induced with 2 % galactose for 4 h, after which 2 % glucose was added to inhibit expression from the *GAL1* promoter. After 0, 2, 4 and 6 h cell extracts were prepared, which were analyzed for heavy (HC) and light chain (LC) content using SDS-PAGE and Western blot. For quantification of IgG signals, the corresponding anti-tubulin signal was used to correct for differences in protein loading. The resulting Western blots are shown in Fig. 6a, c.

**Fig. 7:**
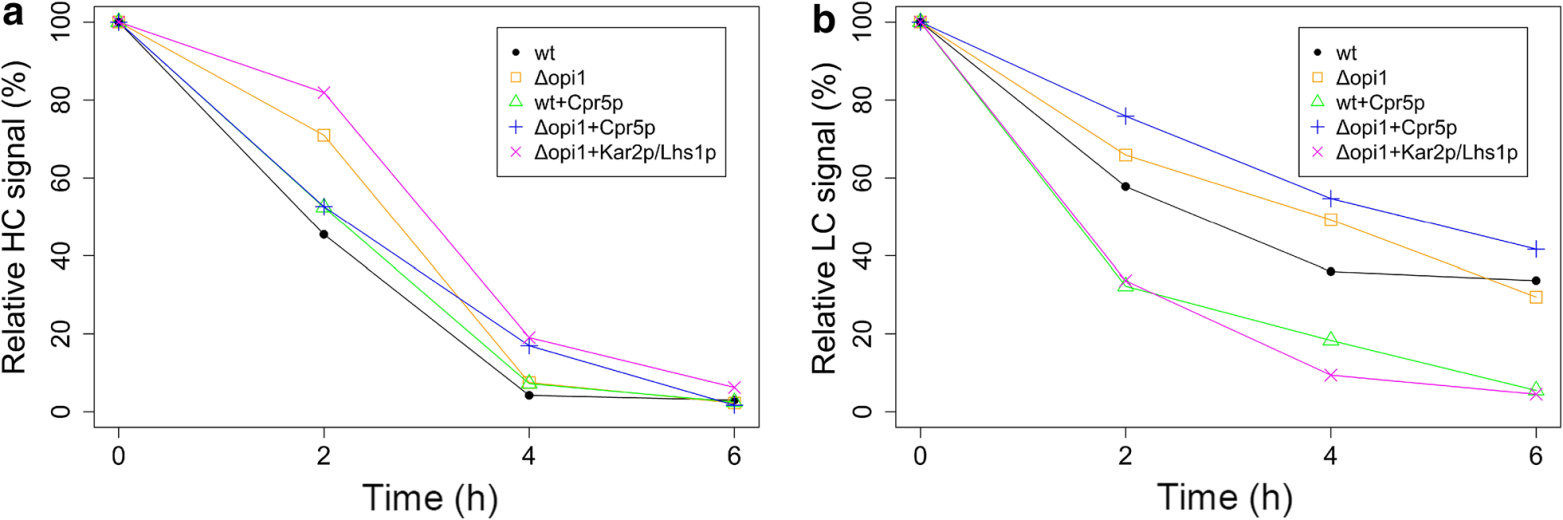
Quantitative analysis of IgG clearance. Signals from the blots (HC and LC) in Fig. 6a and c were quantified and normalized with their corresponding tubulin signals and used to plot their clearance curves in (**a**) and (**b**), respectively. The *data* represents the average of two or three *blots* normalized for loading differences using an anti-tubulin blot. There is a clear variation between HC and LC clearance for all strains and also between the strains in both of the plots. The data gives a general indication that a constant pool of available LC might be beneficial for efficient IgG folding and secretion

With the mock treatment, all strains kept producing IgG for the duration of the assay, as seen by a continuously present signal for the heavy and light chain blots (Control columns of Fig. 6a, c). When IgG expression was repressed by addition of glucose, intracellular IgG levels decreased during the assay and no HC was detected in the samples after 6 h. However, most of the LC samples showed still residual signals at the last time point. Clearance curves were calculated for heavy and light chain that were corrected for loading with the quantified tubulin signals from the glucose treated samples. The relative amount of signal was calculated with respect to the zero time point and the resulting clearance curves are shown in Fig. 7, which shows that HC and LC follow different clearance patterns from each other and between strains. This is not unexpected, as the light chain can be secreted separately, as opposed to the heavy chain that needs the light chain to correctly fold into the full IgG molecule in order to be secreted [21].

For a relative comparison of the intracellular load of HC and LC at the start of the glucose treatment, the samples from zero hours for each strains were compared for HC and LC signal intensities in Fig. 6b, d respectively. A notable difference was the increased intracellular HC concentration in the *Δopi1* derived strains compared with the wild-type derived strains, which was accompanied with a slightly slower clearance. The *Δopi1* strain had an around 75 % higher initial HC load when compared with the wt strain and the best producing *Δopi1* overexpressing Cpr5p strain had more than two times higher HC load compared to wt Cpr5p overexpressing strain. Overall, a trend towards a higher intracellular HC load was detected in the *Δopi1* background. It has been shown for CHO cells that a high cellular content of HC is a molecular marker that characterizes efficient producing clones [62]. The same study showed also a lower correlation between LC content and productivity, while it has been shown that LC content has a strong correlation with strain productivity in heterohybridoma cells [63]. Although the intracellular LC levels in the analyzed best producing *Δopi1* strains are quite similar to wt (Fig. 6d), the clearance of LC is slower in these strains (Fig. 7b), indicating that it might be favorable to have a pool of LC available for full IgG assembly.

The *Δopi1* + *KAR2*-P_GAL1_ + *LHS1*-P_GAL1_ strain, which was showing a decreased IgG secretion, was included in this assay and showed a different pattern from the other strains. Even with a relatively low initial HC (after correction for loading) and LC load compared with the background *Δopi1* strain, the clearance of HC was decreased and the clearance of LC was increased (Fig. 7a, b respectively). These results from our clearance analysis indicate that a constant availability of LC in the cells might be beneficial for an efficient secretion of fully folded IgG molecules. Folding speed might be crucial to improve secretion, but addition of chaperones might have unpredictable effects to the balance between LC and HC, as their function might be fragment specific. Thus, such high-throughput screening methods as presented here are giving insights towards what is needed for effective engineering of multisubunit protein folding.

## Conclusions

We show here that inducing an ER expansion in the yeast *S. cerevisiae* can improve the secretion capacity of full-length antibodies up to fourfold. In addition, six genes (*PDI1, ERO1, KAR2, LSH1, SIL1,* and *CPR5*) were expressed in order to improve the folding environment in the ER. Improving the folding environment by overexpression of ER folding factors is not always achieved in a predictable manner. However, in general the effects of cell engineering on IgG titers are manifesting themselves most clearly at higher cultivation temperatures. Our data demonstrates that expression levels are crucial, especially when combining different factors within one strain, as these showed no synergistic effects in antibody titers. However, by using UPR controlled promoters, the folding environment dynamically responds to changes in ER load and expression levels are adjusted only when necessary, and this could provide a better approach for strain engineering.

The PPIase Cpr5p was shown to enhance antibody secretion the most. Combined with the ER expansion by *Δopi1*, the improved specific antibody yield of over 10-fold higher compared to wild-type was achieved in small scale conditions. Although the strain reached a lower cell density, even the absolute antibody titer was over fivefold higher than the wild-type strain. This makes the *Δopi1* + *CPR5*-P_GPD_ strain a very interesting candidate for further studies, as the combination of strain engineering with process optimization might develop *S. cerevisiae* systems into a viable alternative for current antibody producing platforms.

## Methods

All used FastDigest—restriction enzymes Phusion High-Fidelity DNA Polymerase were obtained from Thermo Fisher Scientific (Vantaa, Finland). All media components and reagents were obtained from Sigma-Aldrich (Helsinki, Finland), unless stated otherwise. Yeast nitrogen base without amino acids (YNB) was obtained from BD (Vantaa, Finland). All sequences of oligonucleotides are listed in Additional file 6.

### Yeast strains

All *S. cerevisiae* yeast strains were derived from the parental strains W303α (ATCC^®^ 208353™). Two types of stable yeast strains were used in this study: W303α and W303α∆*opi1,* both with IgG1-producing genes (heavy and light chains encoding the monoclonal antibody C2B8) integrated to HIS3-site of the genome. These IgG-producing W303α and W303α*∆opi1* background strains are referred to in the manuscript as wt and *∆opi1*, respectively. The W303α∆*opi1* deletion strain was created as described in Hegemann and Heick [64]. A loxP–kanMX–loxP disruption module targeting the *OPI1* gene was generated from pUG6 [65] with PCR, using oligonucleotides 3 and 4. The cassette was transformed to W303α and positive transformants were selected using YPD-agar media, supplemented with 200 µg/mL G418 (Sigma-Aldrich, Helsinki, Finland). Positive knockouts were verified with colony-PCR using oligonucleotides 5 and 6. Cre-recombinase was expressed from plasmid pSH47 [65] to remove the gene disruption cassette from the genome following the protocol presented in Hegemann and Heick [64].

The plasmid for the IgG gene integration was achieved as follows: a fragment from pEK5 plasmid [12], containing genes for heavy and light chain each under GAL1-promoter, was amplified with PCR by using the oligonucleotides 1 and 2. These oligonucleotides contained complementary 5′ ends to the pRS303N-plasmid [66]. pRS303N was linearized with *Ecl*136II and the amplified DNA fragmented was joined with the vector as described in Koskela and Frey [67]. The 6.8 kb *Dra*III and *Bsp*119I fragment of the resulting pEK12 plasmid was transformed into W303α and W303α∆*opi1* strains with the lithium acetate method. Selection of transformed colonies was achieved by adding 200 µg/mL LEXSY NTC (Jena Bioscience, Jena, Germany) to the growth medium. IgG expression of the colonies was confirmed with ELISA.

### Yeast cultures for growth curves

For recording of growth curves, cultures of exponentially growing cells were diluted to OD_600_ of 0.02 in YPD and YPGal (1 % yeast extract, 2 % peptone, and 2 % glucose or galactose, respectively) and liquid synthetic drop out (SD) medium containing 2 % raffinose or 2 % raffinose supplemented with 2 % galactose (SGal). One hundred microliters of these cultures were grown in a round-bottom microtiter plate, with continuous orbital shaking (425 rpm, 3 mm) at 30 °C in an Eon Microplate Spectrophotometer (BioTek, Winooski, USA). OD_600_ measurements were taken every 15 min for 40 h. The growth curves were analyzed with R-package grofit and the characteristic growth parameters were extracted from the spline fit, a data fitting function provided by the grofit-package [68]. The experiment was conducted with seven or eight culture replicates and the statistical significance of the difference between the two strains was determined with Wilcoxon signed rank test (as described below).

### Analysis of the UPR induction

The UPR-responsive cassette encoding GFP from plasmid pDEP017 [35] was linearized with *EcoRV* and integrated into the *TRP1*-locus of *Δopi1* and wt production strains. The functionality of the integrated cassette was confirmed by induction of UPR through addition of 5 mM DTT. For UPR measurements, the strains were grown over night at 30 °C, 250 rpm and diluted to OD_600_ = 0.1 into 96-well plates using SGal media. Cultures were grown with continuous orbital shaking (425 rpm, 3 mm) at 30 °C using a Cytation 3 Microplate Spectrophotometer (BioTek, Winooski, USA), OD_600_ and GFP-fluorescence measurements were taken every 15 min for 24 h.

### Cloning of overexpression plasmids

The genomic DNAs of the selected genes were cloned into yeast shuttle vectors pRS415 and pRS416 containing TEF, GPD and GAL1 promoters [69, 70]. The pRS41X vectors were modified to include the UPR-controlled promoters from *S. cerevisiae* genes *PDI1* and *KAR2*. For exchanging of the promoter, fragments 355 bp upstream of *PDI1* and 281 bp upstream of *KAR2* were first amplified with PCR from yeast genomic DNA, using oligonucleotides 7, 8, 9 and 10, respectively. Next, to generate complementary sequences overlapping with the vector backbone ends for insertion of these fragments with exonuclease and ligation-independent cloning (ELIC) [67], a second round of PCR was performed on these products with oligonucleotides 11, 12, 13 and 14, respectively. These fragments were inserted into *Sac*I/*Xba*I linearized plasmids pRS415 and pRS416 to create the P_PDI1_ and P_KAR2_ empty vectors with the different auxotrophic selection markers.

The plasmids expressing *PDI1*, *ERO1*, *CPR5* and *KAR2* under TEF, GPD and GAL1 promoters, were constructed using PCR on *S. cerevisiae* genomic DNA with their respective oligonucleotide pairs (oligonucleotides 15–22). The PCR amplified fragments were inserted into the *Spe*I *Xho*I sites of the pRS415 and pRS416 vectors, using standard ligation cloning.

The *PDI1*, *ERO1*, *CPR5* and *KAR2* plasmids with P_KAR2_ and P_PDI1_ promoters were constructed by using ELIC. A PCR reaction was made with oligo 36 (3′ end) and the oligo corresponding with the inserted gene from oligonucleotides 23–25 (5′ end), and using the previously created TEF-promoter-gene plasmids as template. The newly generated fragments were used for a second round of PCR to add the complementary sequences for the vectors to the inserts, using oligo 36 (3′ end) with oligo 34 or 35 (5′ end). These final PCR products were inserted into the *Spe*I*/Xba*I and *Xho*I sites of the pRS-shuttle vectors using ELIC.

To construct all the *LHS1* and *SIL1* expression plasmids, oligonucleotides 26&27 and 28&29, respectively, were used to amplify the DNA of *LHS1* and *SIL1* from *S. cerevisiae* genomic DNA. In a second PCR round, the complementary sequences overlapping with the vectors were added to the inserts, using oligo 30 (3′ end) with either oligo 31, 32, 33, 34, or 35 (5′ end). These PCR products were inserted into the *Spe*I*/Xho*I and *Xba*I/*Xho*I fragments of the pRS-shuttle vectors using ELIC cloning.

Empty marker plasmids for use as a control were the recirculated forms of pRS41X-vectors after *Sac*I and *Nae*I digestion. A list of all plasmids used can be found in Additional file 7.

### Yeast expression cultures

Screening of the yeast strains was conducted with 96- deep well plates in a culture volume of 1 ml per well. A colony from each strain was inoculated to SD medium lacking leucine and uracil, containing 2 % raffinose. As a control, the IgG expressing wild-type and ∆*opi1* strains with empty plasmids were included in every plate. Pre-cultures were grown at 30 °C, 250 rpm for 21 h. The culture was diluted 1:4 with fresh SD media, containing 2 % raffinose, supplemented with 0.05 mg/ml BSA. The cultures for IgG expression were grown at 30 °C, 250 rpm) for 5.5 h before protein expression was induced by adding 0.5 or 2 % galactose. Protein expression was continued for 24 h at 20, 25 or 30 °C, 250 rpm. Culture deep-well plates were centrifuged at 2250 rcf, 10 min to clarify the supernatant samples. Cell-free supernatant was divided into two 300 µl samples adjusted to 1x PBT (PBS (135 mM NaCl, 2.5 mM KCl, 10 mM Na_2_HPO_4_, 1.75 mM KH_2_PO_4_) + 0.5 % Tween-20) and stored at −20 °C until analysis, done within 2 days. Each sample was measured with three independent biological replicates, each with two technical replicates in ELISA. OD_600_ was measured before dilution and from final samples with an Eon Microplate Spectrophotometer (BioTek, Winooski, USA).

### Enzyme-linked immunosorbent assay (ELISA) for antibody titer measurement

The ELISA assay was realized on MICROLAB STAR Liquid Handling workstation (Hamilton, Bonaduz, Switzerland) extended with ELx405 Select deep well washer (BioTek Winooski, USA) and Synergy 2 plate reader (BioTek Winooski, USA). Antibody titer was determined as described in de Ruijter and Frey [12].

### Data processing and statistical analysis

All measurement data was collected, combined and moved into R software environment and processed and visualized with the packages therein. Outliers resulting from measurement errors were manually removed after visual analysis of replicate measurements. The performance of the strains were validated with frequency analysis, where the count and relative frequency inside the percentile was determined by strain and for individual measurement points. The cut-off values for 5th and 95th percentiles were determined with quantile() –function. P values are from independent two-group and two-tailed Wilcoxon signed rank test (performed with wilcox.test() –function), where the measurement values of the respective strain background standard served as the reference. The linear regression plane was fitted to the data with the function lm() where dependency of normalized antibody titers on the joint effects of normalized OD_600_ and normalized specific product yield was modeled.

### Real-time PCR

Cultivation was conducted as described for screening assay: Selected strains were inoculated to 1 ml media in 96-deep well plates grown once to saturation and diluted and grown for 5.5 h. After induction with 2 % galactose, the cultures were grown for 2 or 10 h at 30 °C. 1.5 OD_600_ of cells was collected for mRNA isolation. IgG expression was confirmed from supernatants with ELISA. mRNA was isolated from cell pellets with RNeasy^®^ Mini Kit (Qiagen), and approximately 10 µg of mRNA was treated with TURBO DNA-free™ DNAse (Ambion^®^ RNA by Life Technologies). After DNAse inactivation, mRNA was converted to cDNA with AffinityScript QPCR cDNA synthesis kit (Agilent Technologies, USA), according to manufacturer’s protocol. qPCR was performed with a CFXConnect thermocycler (Bio-Rad, USA) on 100 ng of cDNA using commercial PrimePCR™ SYBR^®^ Green Assay CPR5, Yeast primers (qSceCED0004208, Bio-Rad, USA), while *ACT1* levels were measured as a loading reference with primers 37 and 38. Maxima SYBR green/fluorescin qPCR Master Mix (Thermo Scientific, USA) was used as reagent according to manufacturer’s protocol. Results from three technical replicates were analyzed with the Bio-Rad CFX Manager 3.0 software, using *ACT1* as loading control and with normalization to the *CPR5* signal from the wt sample.

### Cellular clearance assay

The cellular clearance assays was performed as described in De Ruijter and Frey [12]. Using anti-human IgG (Fc specific)-peroxidase labelled antibody produced in goat for heavy chain detection and anti-Human kappa light chains (Bound and Free)-peroxidase antibody produced in goat for light chain detection. Rabbit anti-tubulin antibody [EPR13799] (Abcam, Cambridge, UK) and goat anti-rabbit IgG (Fc specific)-peroxidase labelled were used for tubulin detection. Signal detection was done with the Supersignal™ West Pico Chemiluminescent Substrate (Thermo Fisher Scientific, Vantaa, Finland), following the manufacturer’s instructions.

## Additional files

10.1186/s12934-016-0488-5 Results from the screening process for all the strains with single gene additions, divided by the different experimental conditions. Values without defined gene or promoter are the strain background measurements for each conditions. The normalization of samples is always done to the background strain values from the same condition. Specific product yield is defined as antibody titer (denoted [AB]) divided by OD_600_-value. SD is an abbreviation for standard deviation. The values are means from three biological and two technical replicates.
10.1186/s12934-016-0488-5 Effects of single folding factor overexpression on antibody secretion in wt and Δ*opi1* strains at 2 % galactose induction. A heatmap illustrating the effects that the different individual elements had on specific product yields in wt (A) and the Δ*opi1* strain (B) backgrounds at 2 % induction at 30 °C. The numbers represent the fold-changes that are calculated by normalizing to the respective mean specific product yields of the wt and Δ*opi1* background strains.
10.1186/s12934-016-0488-5 Screening results for all the strains with double gene additions grouped by strain background, temperature and induction strength. First, the values for the background strain standards are listed, to which the normalization is done always using the reference from the same condition. [AB] stands for antibody titer and SD for standard deviation and specific product yield is defined as [AB]/OD_600_. The values are means from three biological and two technical replicates.
10.1186/s12934-016-0488-5 Overview of the mean fold-changes for all the strains with double gene additions. For each analyzed gene pair the mean fold-changes in OD_600_, in IgG titer, and in specific product yield are shown. Averages were calculated from fold-changes that were first compared to the respective wild-type samples.
10.1186/s12934-016-0488-5 Result from the frequency analysis showing the strains in the 95th and 5th percentile of individual measurements of specific product yield in each temperature and strain background. To determine which strains were enriched among the highest and lowest values of specific product yield, we conducted a frequency analysis for each strain background, separately for each temperature. Frequency represents the absolute count of measurements from the strain inside the percentile and the relative frequency is the frequency normalized to the total count of measurements in the percentile. P values were calculated with Wilcoxon signed rank test using the respective background strain measurements in the same temperature as the reference.
10.1186/s12934-016-0488-5 List of oligonucleotides used in this study. All sequences of oligonucleotides used in this work are shown. Oligonucleotides 1–6 are for DNA integration into the yeast genome and for its confirmation. 7–14 were used for placement of P_PDI1_ and P_KAR2_ into the vector backbone, 15–36 were used for generation of the overexpression plasmids of the various folding factors. Finally 37 and 38 were used for *ACT1* qPCR.
10.1186/s12934-016-0488-5 List of all the plasmids in our overexpression library. Overview of all ER-resident protein expression plasmids used in this study. Original name, promoter, expressed gene and the corresponding auxotrophic marker are mentioned. All plasmids contain the cyc1 terminator.
