# Supplementary figures and images for: Enhancing antibody folding and secretion by tailoring the Saccharomyces cerevisiae endoplasmic reticulum
Source: Microb Cell Fact. 2016 May 23;15:87. doi: 10.1186/s12934-016-0488-5 (PMC4878073; doi:10.1186/s12934-016-0488-5)

A

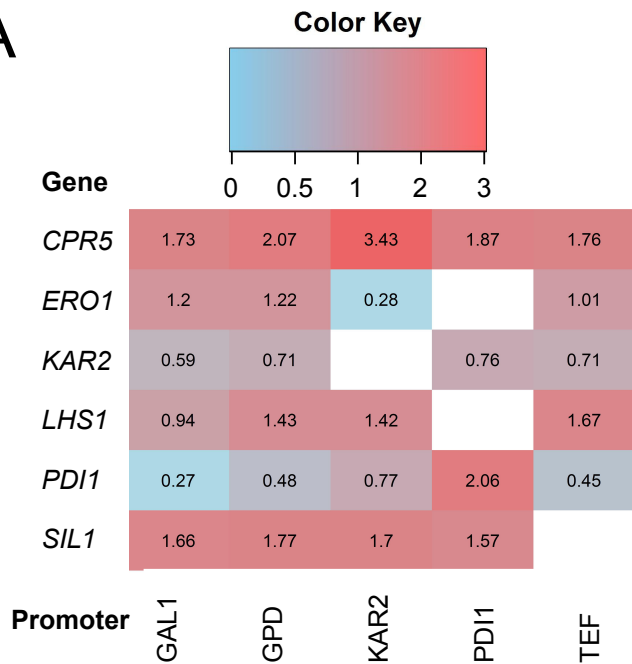

B

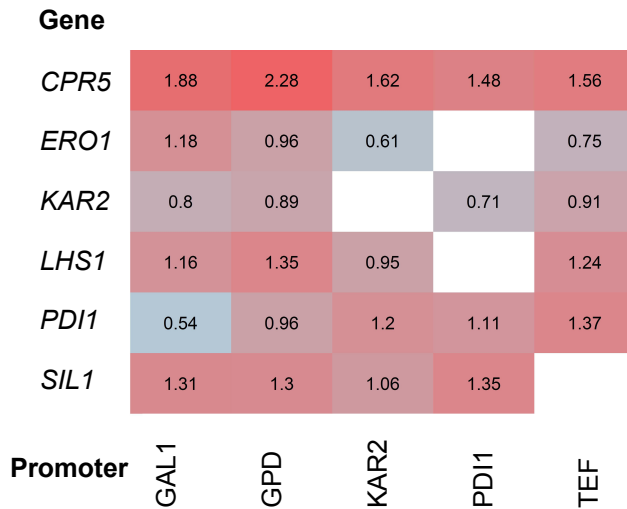

Supplement: Supplementary file 2 — 10.1186/s12934-016-0488-5 Effects of single folding factor overexpression on antibody secretion in wt and Δopi1 strains at 2 % galactose induction. A heatmap illustrating the effects that the different individual elements had on specific product yields in wt (A) and the Δopi1 strain (B) backgrounds at 2 % induction at 30 °C. The numbers represent the fold-changes that are calculated by normalizing to the respective mean specific product yields of the wt and Δopi1 background strains. [file 12934_2016_488_MOESM3_ESM.pdf]
